# Adaptive plasticity and niche expansion in an invasive thistle

**DOI:** 10.1002/ece3.1599

**Published:** 2015-07-14

**Authors:** Kathryn G Turner, Hélène Fréville, Loren H Rieseberg

**Affiliations:** 1 Department of Botany and Biodiversity Research Centre, University of British ColumbiaRoom 3529-6270 University Blvd., Vancouver, British Columbia, V6T 1Z4, Canada; 2 UMR 1334 AGAP INRA2 place Pierre Viala, Montpellier Cedex 2, 34060, France; 3 UMR 5175 CEFE CNRS1919 route de Mende, Montpellier Cedex 5, 34293, France; 4 Department of Biology, Indiana UniversityBloomington, Indiana, 47405

**Keywords:** Adaptive plasticity, biological invasions, *Centaurea diffusa* (diffuse knapweed), climatic niche, evolution of invasive species, trade-offs

## Abstract

Phenotypic differentiation in size and fecundity between native and invasive populations of a species has been suggested as a causal driver of invasion in plants. Local adaptation to novel environmental conditions through a micro-evolutionary response to natural selection may lead to phenotypic differentiation and fitness advantages in the invaded range. Local adaptation may occur along a stress tolerance trade-off, favoring individuals that, in benign conditions, shift resource allocation from stress tolerance to increased vigor and fecundity and, therefore, invasiveness. Alternately, the typically disturbed invaded range may select for a plastic, generalist strategy, making phenotypic plasticity the main driver of invasion success. To distinguish between these hypotheses, we performed a field common garden and tested for genetically based phenotypic differentiation, resource allocation shifts in response to water limitation, and local adaptation to the environmental gradient which describes the source locations for native and invasive populations of diffuse knapweed (*Centaurea diffusa)*. Plants were grown in an experimental field in France (naturalized range) under water addition and limitation conditions. After accounting for phenotypic variation arising from environmental differences among collection locations, we found evidence of genetic variation between the invasive and native populations for most morphological and life-history traits under study. Invasive *C. diffusa* populations produced larger, later maturing, and therefore potentially fitter individuals than native populations. Evidence for local adaptation along a resource allocation trade-off for water limitation tolerance is equivocal. However, native populations do show evidence of local adaptation to an environmental gradient, a relationship which is typically not observed in the invaded range. Broader analysis of the climatic niche inhabited by the species in both ranges suggests that the physiological tolerances of *C. diffusa* may have expanded in the invaded range. This observation could be due to selection for plastic, “general-purpose” genotypes with broad environmental tolerances.

## Introduction

Much recent research in invasion biology has assessed whether populations of invasive plants show heritable phenotypic differences in growth and reproduction between their native and invaded ranges, in an effort to understand the causal drivers of invasion (Thébaud and Simberloff 2001; Hinz and Schwarzlaender 2004; Bossdorf et al. 2005; Felker-Quinn et al. 2013). Where such differences are not found, species that successfully invade may be preadapted, that is, already well suited to the typically anthropogenically disturbed conditions found in the novel habitat. Such preadaptation may result from prior adaptation (defined here as a heritable selection-driven change in phenotype that increases fitness) to frequent disturbance or human-altered habitats in the native range (Lee and Gelembiuk 2008; Hufbauer et al. 2012; Mráz et al. 2012b; Foucaud et al. 2013). Indeed, a species is more likely to establish a self-sustaining population in a new location if there is at least some degree of environmental overlap with the native range (Bock et al. 2015). Yet invasion success may depend on the capacity of a species to adapt to novel environmental conditions, and rapid adaptive change has been documented in many invasive species (reviewed in Dlugosch and Parker 2008; Felker-Quinn et al. 2013), often occurring over very short time spans (Whitney and Gabler 2008; Buswell et al. 2011). This rapid evolution is often understood to be the result of environmental differences between the ranges generating strong selective pressures (Bock et al. 2015).

Clinal, genetically based, phenotypic variation demonstrated among invasive populations represents some of the best evidence for rapid evolution in the invaded range, including adaptation to latitudinal and altitudinal clines (Alexander et al. 2009; Bock et al. 2015), although multiple introductions and admixture may play an underappreciated role in driving clinal variation (Kao et al. 2015). Local adaptation (defined here as a change in allele frequencies leading, on average, to higher relative fitness in a population’s local habitat than genotypes originating from other habitats, in other words, specialization in local habitats; Kawecki and Ebert 2004) can quickly shape phenotypic variation during range expansion along selective climate gradients as the invading populations adjust to local environments, shifting phenology, biomass, and other trait means (Colautti et al. 2010). Such local adaptation along selective gradients can cause rapid evolution during invasion and may have a stronger effect on the fitness of an invasive species than enemy release or the evolution of increased competitive ability (EICA) (Colautti and Barrett 2013; Zenni et al. 2014a).

Several other evolutionary hypotheses invoke trade-offs in resource allocation to account for genetically based phenotypic differences between the native and invaded ranges. A trade-off occurs when a beneficial change in one trait is opposed by a detrimental, concomitant change in a second trait (Roff and Fairbairn 2007). If novel habitats are less stressful, either biotically, for example, due to the absence of specialist herbivores (EICA; Blossey and Notzold 1995; Joshi and Vrieling 2005), or abiotically, for example, when resources are abundant (Bossdorf et al. 2005; He et al. 2010), selection would favor individuals that shift resource allocation from stress tolerance to increased vigor and fecundity and, therefore, invasiveness. Such trade-offs and their role in the invasion process have been assessed by several studies (Hodgins and Rieseberg 2011; Lachmuth et al. 2011; Kumschick et al. 2013; Turner et al. 2014), but these attempts are complicated by the variability of favored strategies between different habitats (Lachmuth et al. 2011).

Rather than specialize in local novel environments through a micro-evolutionary response, invasive species may instead benefit from generalist strategies, whereby the plastic responses of a “general-purpose” genotype may confer fitness advantages in many environments (Baker and Stebbins 1965; Richards et al. 2006). Phenotypic plasticity refers to the potential of specific traits of a genotype to respond to different environments; adaptive phenotypic plasticity in fitness traits enhances an organism’s survival and reproduction across different environments (Richards et al. 2006) and has been confirmed in some weedy plant species (Hahn et al. 2012; Zenni et al. 2014b; Bock et al. 2015). Phenotypic plasticity that enables fitness homeostasis, permitting a genotype to adjust its phenotype to maintain fitness even in stressful or unfavorable environments, is known as the “Jack-of-all-trades” strategy (Richards et al. 2006).

Theoretical work suggests that while stable environments will favor local adaptation, frequent and unpredictable disturbance (e.g., anthropogenic disturbance) will rapidly select for potentially invasive, phenotypically and developmentally plastic genotypes, in either the native or invaded ranges, well suited for colonizing novel habitats (Meyers et al. 2005; Hufbauer et al. 2012). Extreme environmental changes, such as those experienced in a novel habitat, can result in the rapid evolutionary increase in plasticity, although this may be a transient effect if plasticity is costly to maintain (Lande 2009, 2015). Plasticity may enhance ecological niche breadth because plastic responses may allow a species to express advantageous phenotypes in a broader range of environments (Richards et al. 2006; Hahn et al. 2012; Zenni et al. 2014b). Yet at least among Holarctic invasive plants, evidence of species persisting in climatic environments outside of those experienced in the native range, that is, shifts in the realized climatic niche, potentially enabled by increased plasticity, is rare (Petitpierre et al. 2012; but see Webber et al. 2012).

Here, we report on a field-based common garden study of genetically based phenotypic differentiation between native and invasive populations of *Centaurea diffusa* (diffuse knapweed), one of the North America’s most problematic weedy invaders (Lejeune and Seastedt 2001). Phenotypic differentiation was demonstrated in two previous glasshouse common garden experiments, which compared phenotypes of 57 populations of native and invasive *C. diffusa* under benign and stressful conditions, including drought, flood, nutrient deficiency, and herbivory (Turner et al. 2014). Increased fitness in invasive populations as a result of phenotypic differentiation would represent a possible causal driver of invasion. Such phenotypic differentiation could be due to local adaptation to new environmental conditions experienced among populations in the invaded range (Hypothesis 1a) and may reflect a shift in resource allocation that results in a trade-off (Hypothesis 1b). Alternately, phenotypic differentiation from the native range could be the result of the evolution in the invaded range of a plastic, environmental generalist, strategy that maintains fitness homeostasis under a wide range of conditions (Hypothesis 2). To distinguish between these two hypotheses, we examine phenotypic differences between populations from the native and invaded ranges, using a common garden experiment in the naturalized range of *C. diffusa* in Montpellier, France, to test for evidence of local adaptation or increased phenotypic plasticity. The naturalized range represents an area known to be within the physiological tolerances of *C. diffusa* (it is reported there, though rarely; Greuter 2009), and yet external to both the native and invaded ranges. If trait divergence is at all due to local adaptation, then investigating performance within either range could favor local populations if individuals experience biotic or abiotic conditions more typical of their “home range” (Colautti et al. 2009). Thus, our experimental design allowed us to examine performance under more realistic field conditions (as compared to the previous glasshouse study from Turner et al. 2014), while minimizing potential “home range” biases.

We investigate evidence for local adaptation to environmental conditions (Hypothesis 1a) by measuring phenotypes in a common environment of plants sampled from native and invaded ranges from a variety of environmental conditions. Further, we test whether patterns of genetically based phenotypic variation are in agreement with a resource allocation trade-offs (Hypothesis 1b) by comparing plant traits from both ranges in the presence or absence of experimentally applied water addition and limitation, a trade-off implicated in a previous study (Turner et al. 2014). We test whether invasive populations of *C. diffusa* perform better than native populations in a field setting in the naturalized range and whether performance is correlated with phenotype under water limitation. If resource shift along a trade-off between drought tolerance and growth rate is a causal driver of invasion, then invasive populations should perform significantly more poorly than native populations under water limitation. In the absence of local adaptation (including trade-offs), selection for increased phenotypic plasticity in the invaded range may instead explain the spread and dominance of invasive populations over many habitats (Hypothesis 2). Therefore, using publically available occurrence data, we further examine the climate space inhabited by native and invasive *C. diffusa* at a larger spatial scale than our sampled populations, to test the prediction that plasticity in environmental tolerance should expand the realized climatic niche in the invaded range of *C. diffusa*.

## Materials and Methods

### Study species

Within a large family containing many weeds (Asteraceae), the genus *Centaurea* has contributed 30 nonnative species to North America, including 11 noxious weeds (USDA 2014), and is one of the only 15 plant genera in the United States to contain more weedy species than expected by chance (Kuester et al. 2014). The five *Centaurea* species with the greatest impact, including *Centaurea diffusa* Lam. (diffuse knapweed), have invaded millions of hectares of grassland, making it the most abundant noxious weed genus in the western United States (Lejeune and Seastedt 2001). *Centaurea diffusa* is typically a monocarpic, facultative biennial (Thompson and Stout 1991), which forms a basal rosette and then bolts and dies after reproducing.

**Figure 1:**
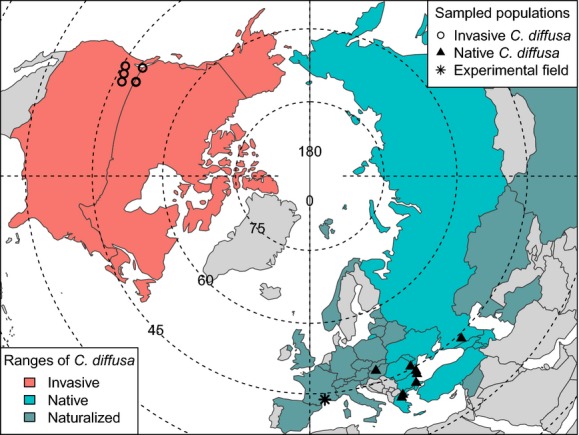
Range and population map of *Centaurea* in the Northern Hemisphere, by country, used in the field experiment. Origin of sampled population (invasive or native *C. diffusa*) is indicated by point shape. Origin status in each country is indicated by color. Degrees of latitude are indicated on dotted lines and longitude on solid lines. Modified with permission from Turner et al. (2014).

Native to parts of eastern Europe and western Asia, *C. diffusa* is found sparsely throughout western Europe, where it is considered a naturalized alien (Fig.1; Greuter 2009; Bleeker et al. 2007). First reported in North America more than 100 years ago (Sheley et al. 1998), it now occurs in roughly half of Canada and the United States (Fig.1; USDA 2014). Surveys of genetic diversity in this species suggest that (1) *C. diffusa* has been introduced to North America multiple times (at least once from Turkey); (2) comparable genetic diversity exists within each range; and (3) little population structure is evident in the native range (Hufbauer and Sforza 2008; Marrs et al. 2008).

### Populations

Seeds were collected in a broad collaborative effort from eight native European populations and six invasive North American populations of *C. diffusa* as part of a large sampling scheme of Turner et al. (2014). Collection dates ranged from 2005 to 2010, with the majority of populations collected in 2008 (Table S1).

### Common garden experiment

To compare the phenotypes of invasive *C. diffusa* to native *C. diffusa* and look for evidence of local adaptation (Hypothesis 1a and Hypothesis 1b) or increased fitness homeostasis (Hypothesis 2) in the invaded range, we measured phenotypes in a common environment. In the spring of 2011, we initiated a field common garden in the naturalized range of *C. diffusa* at Montpellier (CEFE, Plateforme des Terrains d’expériences du Labex CeMEB), Languedoc-Roussillon, France, near the northern edge of the Mediterranean Sea. Seeds collected from four to six mothers at each of 14 collection locations were used (Table S1). In total, this common garden included 263 individuals, with a mortality rate of 14%, so that 225 survived until harvest.

Seeds were germinated on filter paper in distilled H_2_O in a temperature-controlled chamber which was maintained to a diurnal range of 12–22°C. Supplemental lighting provided a 16 h day. Within 15 days, *c*. 400 seedlings were transplanted into 8-cm-square peat pots (Jiffy Products International B.V., Moerdijk, the Netherlands) filled with 33% tomato potting mix, 33% silica sand, and 33% steam-sterilized field soil. Seedlings were grown in climate-controlled glasshouse and watered daily. When plants reached the median eight-leaf stage (4 week after germination), they were transplanted to the field.

Individuals were planted into an experimental field in a randomized block design, with 0.5 m between rows and between plants within a row and each row assigned a treatment. Plants from each population were randomly assigned to a treatment. Treatments included an irrigated water addition control and nonirrigated water limitation treatment. Because water limitation tolerance was only a subset of one of our hypotheses (1b), roughly twice as many plants were assigned to the control group than to the water limitation treatment. Nonexperimental plants were planted around the edge of the plot to lessen edge effects. After transplantation, all plants received supplemental watering every 12 h. Once treatment began, 2 week after field planting, irrigation to water limitation treatment rows ceased. Thus, these plants only received water from natural rainfall.

Morphometric and life-history measurements were taken several times over the course of 4 months, and these measurements were taken blind. Repeated morphometric measurements were taken before treatment began (2 week after transplantation), during treatment (4 week after transplantation), and at harvest (at bolting or 4 months after transplantation for those plants that did not bolt) and included length and width of longest leaf, number of basal leaves >3 cm long, and maximum diameter of basal rosette. Four weeks after transplantation, a subset of plants (126) were sampled for specific leaf area (SLA); one leaf per plant was harvested, image scanned while fresh, and leaf area calculated using ImageJ (Rasband 2011). Life-history traits were assessed weekly and included bolting probability, bolting date, date of first stress response (wilting or yellowing), and mortality. When a plant bolted, but before it flowered, it was measured and harvested to avoid release of pollen or seed from potentially invasive genotypes. Additional measurements were taken at harvest, including shoot mass, root crown diameter, and approximate rosette area (maximum diameter × perpendicular diameter × *π*/4). The leaf sampled for SLA and the harvested shoot material for each plant were stored separately in paper bags and oven-dried at approximately 65°C for at least 3 days, and then, weight measurements were taken.

### Statistical analysis

To determine how *C. diffusa* differs phenotypically between its native and invasive ranges, we compared morphological and life-history traits among *C. diffusa* individuals. Using R 3.0.1 (R Core Team 2014), we employed restricted maximum-likelihood (REML) models with random effects using the lme4 package. Univariate response traits included root crown diameter, rosette area, shoot mass, SLA, bolting probability, bolting date, date of first wilting, and mortality rate. Repeated measurements of a trait (leaf count, length and width of longest leaf, and rosette diameter) were analyzed together, and measurement date and individual were included as random effects. Gaussian distributions were fit for continuous measures, and trait values were natural-log-transformed when necessary to improve normality of residuals. Poisson distributions were fit for count data, and binomial distributions were fit for binary data. Data were scaled when necessary to improve model performance.

To account for phenotypic variation arising from environmental differences between sampled locations, each full model included a composite abiotic environmental covariate determined by a principal component analysis (PCA) of altitude, latitude, and 19 bioclimatic variables of each sampled seed collection location taken from the WorldClim database of current climatic conditions (hereafter, the “experimental PCA”; Table S2; Hijmans et al. 2005). The principal component that explained the most variance among collection locations (PC1) was used in all trait models as the environment term. Models were also run using a second composite environmental variable (PC2) or only latitude in place of the environment term, but this did not substantially alter results (*not shown*).

To test how invasive populations of *C. diffusa* perform relative to native populations, we ran range differentiation models, where origin (native or invasive), environment (PC1 from the experimental PCA), and their interaction, as well as treatment (water addition or limitation), were included as fixed effects in all full models. Population (uniquely named) and maternal lines nested within population were used as random effects in all full models (e.g., Trait ∼ Origin × Environment + Treatment + (1 ¦ Population/Maternal line)). When a range differentiation model had a significant origin-by-environment term, slopes of regression lines from model estimates are reported. Additionally, to test for resource allocation trade-offs, we assessed differences in morphological and life-history traits between treatments using models that explicitly tested for a trade-off between performance in benign conditions and tolerance to water limitation (Data S1).

To assess the significance of each model term, we removed each term or interaction in a stepwise manner based on likelihood ratio tests (LRTs). All LRTs were corrected for multiple comparisons using the false discovery rate (FDR) procedure implemented in the “qvalue” package v.1.40.0, with an FDR cutoff value of 5% and the “bootstrap” method (Storey et al. 2004). However, because this correction did not change the significance of any fixed effect (and only four random effects of 65 LRTs; Table1, Table S3), and because model terms were included based on the *P* value of each LRT, significance based on *P* value is reported. Chi-squared test statistic, degrees of freedom, and significance (*P* value < 0.05) are reported from these LRTs. When all random effects were nonsignificant, generalized linear models (GLMs) were used, and the results of these LRTs are reported. All non-Gaussian minimal GLMs were checked for overdispersion. For models with significant origin or origin-by-environment term, model estimates are reported for fixed effects. Because the effect of one variable depends on the condition of the other, it is not meaningful to test the significance of main effects that are included in significant interactions during stepwise model simplification (Crawley 2012), and so, these are not reported.

**Table 1:** Test statistics from range differentiation models of phenotypic measurements of *Centaurea diffusa,* for all traits measured in the field experiment with a significant origin or origin-by-environment term

Trait	Fixed effects				Random effects		
	Origin	Env	Origin × Env	Treatment	Population	Maternal line	Repeat measure
	*χ*^2^ (df) *P*	*χ*^2^ (df) *P*	*χ*^2^ (df) *P*	*χ*^2^ (df) *P*	*χ*^2^ (df) *P*	*χ*^2^ (df) *P*	*χ*^2^ (df) *P*
Number of basal leaves^1^	5.82 (1)*	0.49 (1)	0.03 (1)	0.12 (1)	0.81 (1)	0 (1)	552.87 (3)***
Width of longest leaf	nt	nt	8.50 (1)**	0.82 (1)	1.02 (1)	0.85 (1)	132.38 (3)***
Root crown diameter	nt	nt	9.88 (1)**	0.82 (1)	14.89 (1)***	16.33 (1)***	–
Rosette area at harvest	nt	nt	8.35 (1)**	5.23 (1)*	24.23 (1)***	3.16 (1)	–
Shoot mass	nt	nt	14.44 (1)***	1.71 (1)	14.82 (1)***	9.71 (1)**	–
Bolting probability	nt	nt	37.19 (1)***	0.06 (1)	0 (1)	0 (1)	–
Bolt date	nt	nt	9.34 (1)**	0.07 (1)	4.84 (1)*,^2^	0 (1)	–
Wilt date	nt	nt	6.28 (1)*	21.42 (1)***	4.76 (1)*,^2^	0 (1)	–
Yellow date	nt	nt	25.89 (1)***	0.46 (1)	4.45 (1)*,^2^	0 (1)	–

Results are presented from restricted maximum-likelihood (REML) models. Significance of term indicated by symbol

* *P *<* *0.05;

** *P *<* *0.01

*** *P *<* *0.001.

Env, environment term; df, degrees of freedom; *χ*^2^, chi-squared test statistic; nt, not tested because of significant interaction term.

1 Data scaled when necessary to improve model performance.

2 Nonsignificant after FDR correction.

### Occurrence data and principal component analysis

To determine whether differing relationships between phenotype and environment between origins observed in our dataset are reflected in a difference between the realized climatic niches of the species ranges at a spatial scale larger than our sampling area, we investigated evidence of a climatic niche expansion in the invaded range of *C. diffusa*. Five hundred and ninety-two geo-referenced occurrence locations for *C. diffusa* from North America, Europe, and western Asia were retrieved from the Global Biodiversity Information Facility, using the R package “rgbif” (Chamberlain et al. 2014; GBIF 2014). This was combined with 70 seed collection locations from previous sampling efforts (Turner et al. 2014). For each occurrence record, corresponding climate data were retrieved from the WorldClim database as above (Table S2; Hijmans et al. 2005). This dataset was then used in a PCA of the climate, altitude, and latitude of all occurrence locations (hereafter, the “occurrence PCA”). The magnitude and statistical significance of the niche shift between the occurrence centroids in the invaded and native ranges in the PCA graph were assessed using a between-class analysis with the R package “ade4” yielding a between-class inertia percentage (Broennimann et al. 2007; Dray et al. 2007). This ratio was further tested with a Monte Carlo randomization test (999 iterations; Dray et al. 2007). In addition, 99% confidence ellipses describing the cluster for each range using the bivariate t-distribution are presented. Because the GBIF data used here may not be error-free, we reran this analysis using only populations within two standard deviations away from the mean of PC1 and PC2 to verify results.

## Results

### Principal component analysis of sampled populations

**Figure 2:**
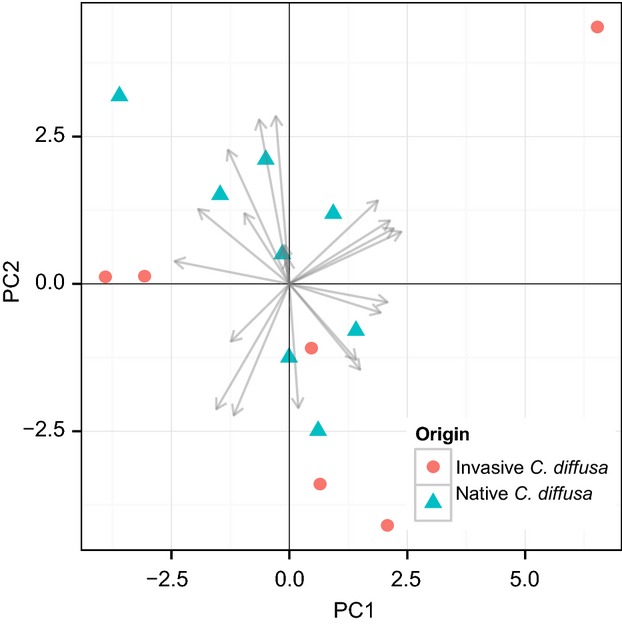
Principal component analysis of abiotic environmental variables of sampling locations of sampled populations of *Centaurea diffusa*. Climate data from WorldClim database (Hijmans et al. 2005). Variables defined in Table S2.

The first two components obtained by the experimental PCA of abiotic environmental variables characteristic of each seed collection location explain 33% and 28% of variance among the collection locations of the sampled populations, respectively (Fig.2, Figure S1). Axis 1 was correlated most strongly with maximum temperature of the warmest month, annual precipitation, and precipitation during the wettest periods (BIO5, BIO12, BIO13, BIO16; Table S2) and can be conceptualized in terms of “aridity,” with small values associated with dry, hot summers. Axis 2 was correlated most strongly with minimum and mean temperature of the coldest periods, annual mean temperature, and temperature seasonality (BIO6, BIO11, BIO1, BIO4) and can be thought of as “harshness of winter,” with small values associated with cold winters. A comparison of invasive and native *C. diffusa* sample locations for these two axes indicates a substantial degree of overlap of climatic niches for these populations (blind 95% confidence ellipses group most populations into a single cluster, Figure S1B). The degree of dispersion among populations may indicate that native populations were sampled from a narrower range of environments. Nevertheless, later analyses had sufficient power to detect significant differences between native and invasive populations and their relationship to an environmental gradient.

### Phenotypic differentiation

**Figure 3:**
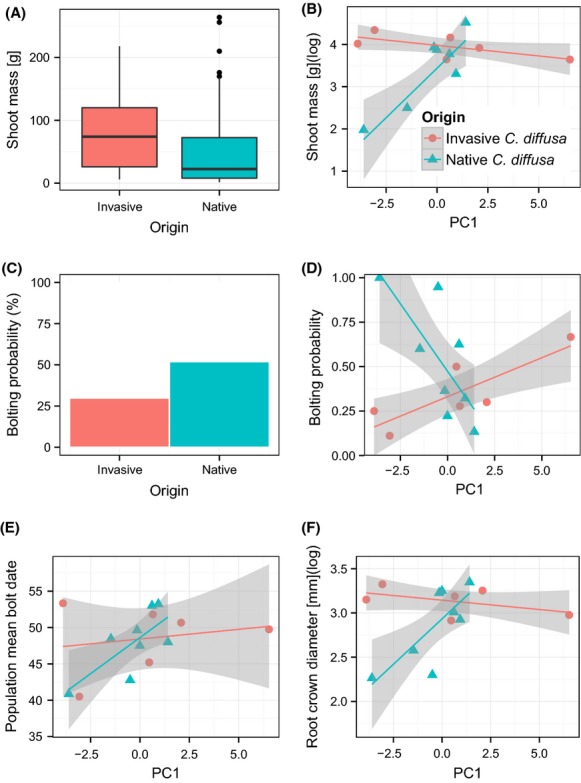
Selected examples of size and life-history trait divergence among *Centaurea diffusa* ranges in the common garden experiment. All figures are from observed data; model parameters are described in Tables1 and S4. Environment is represented in each figure by PC1 of sampled population locations. Shaded area represents standard error. (A) Shoot mass at harvest by origin. (B) Population mean shoot mass (log-transformed) along the environmental gradient (significant origin-by-environment interaction). (C) Proportion of each group which had matured (i.e., bolted) by harvest. (D) Population mean bolting probability along the environmental gradient (significant origin-by-environment interaction). (E) Population mean bolt date, among plants that bolted, along the environmental gradient (significant origin-by-environment interaction). (F) Population mean root crown diameter along the environmental gradient (significant origin-by-environment interaction). In (B), (D), (E), and (F), origin is indicated by point shape; invasive *C. diffusa* as circles, native *C. diffusa* as triangles.

In the common garden dataset, of the 13 traits assessed for range differentiation, nine exhibited significant phenotypic differentiation between the native and invaded ranges of *C. diffusa* (leaf number, leaf width, root crown diameter, rosette area, shoot mass, bolting probability, bolt date, wilt date, and yellowing date; Table1, Table S3; for correlation among traits, see Figure S2). In each case, origin (native versus invasive) had an effect on trait values, often via an interaction, but sometimes not. Origin was significant for leaf number and was marginally significant for leaf width and rosette diameter. Random effects were common, and at least one (most commonly population; Table1) was significant in every model that differentiated the two ranges, except bolting probability. Specific leaf area and mortality rate did not differ significantly between treatments or ranges. For every measure of size which varied significantly or marginally significantly between the two ranges, invasive individuals were larger (Table S4, Fig.3A, B and F, Figure S3). For example, invasive rosettes had approximately 32 grams more shoot mass than natives in the control treatment (observed means and standard errors: invasive 83.96 ± 7.66, native 51.46 ± 6.27, Fig.3A and B). All size traits with a significant origin-by-environment term displayed a similar trend: For invasive populations, size did not significantly vary with environment, whereas for native populations, size significantly changed along the environmental gradient (increase: root crown diameter, rosette area, shoot mass; decrease: leaf width; Table S4, Figure3F, Figure S3). In other words, for native populations, the hotter and drier the climate experienced at the source location, the smaller the individual a population produced. Leaf width is an interesting exception to this trend; leaf width in native populations appears to decrease along the environmental gradient for the first two time points, but it increases during the third, resulting in an overall negative slope (Figure S3, Table S4). Invasive populations have lost the relationship to this environmental gradient. Life-history traits also differentiated the two ranges; invasive individuals were less likely to bolt during the course of the experiment (observed mean and standard error in control treatment: invasive 29.5 ± 5.9%, native 52.4 ± 5.5%; Fig.3C and D). Both native and invasive individuals exhibited a significant relationship to the environmental gradient for bolting, although in the opposite direction. Moving along the environmental gradient (PC1) toward wetter climates with milder summers, native individuals decreased their probability of bolting, whereas invasive individuals increased their probability of bolting. Although the subset of plants which bolted during the course of the experiment was less than half (28 invasive and 68 native individuals), there was a significant interaction between origin and environment for bolt date, such that the milder and wetter the climate experienced by native populations, the later the bolting date, while bolting date in invasive individuals had no significant relationship to the environmental gradient of collection location (Fig.3E).

### Water limitation response and resource allocation trade-offs

Only two traits demonstrated a significant effect of treatment in range differentiation models (rosette area, date of wilting), suggesting a limited impact of water addition (Table S3). Explicit trade-off models of water limitation treatment plants revealed no significant interactions between origin and population mean performance in the benign control treatment for any trait (*not shown*). Total natural rainfall at the field location during the duration of the water limitation treatment (June–September) was 170.4 mm, but water addition to the experimental plot was not directly measured.

### Evidence of niche expansion

**Figure 4:**
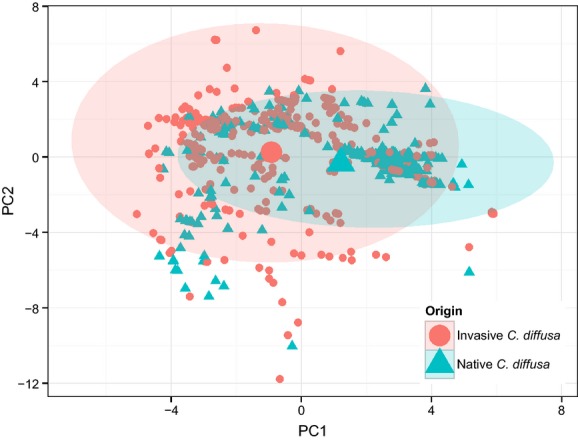
Putative climatic niche expansion as determined by principal component analysis of occurrence data in the native and invasive ranges of *Centaurea diffusa*. Shaded area represents 99% confidence ellipse for each range. Centroid of niche marked by large point.

Principal component analysis of the climatic data of all *C. diffusa* occurrences (376 invasive and 286 native occurrences) in the occurrence dataset defined the realized environmental space by two significant axes of variation. The first two components obtained by the occurrence PCA of abiotic environmental variables explained 32% and 27% of variance among occurrences, respectively (Fig.4, Figure S4). Axis 1 was correlated most strongly with precipitation during driest month and quarter/mean diurnal temperature range (BIO14, BIO17, BIO2; Table S2). Axis 2 was correlated most strongly with precipitation during the coldest and wettest quarters, and mean temperature during the coldest quarter (BIO19, BIO16, BIO11). Niche centroids in this dataset differ slightly but significantly between ranges (between group inertia: 6.85%; *P* = 0.001). Although the 99% confidence ellipses of the invaded range covers most of the climate space in the native range ellipse, evidence suggests that the invaded niche has shifted into more arid climates (toward lower values of PC1) and expanded into habitats with a broader range of precipitation during cold and wet periods (expanded in both directions along PC2; Fig.4). After subdividing the data to include only populations within two standard deviations of the mean of PC1 and PC2 (613 populations), the pattern of putative range shift and expansion remained (Figure S5), and niche centroids differed slightly more (between group inertia: 9.31%; *P* = 0.001).

## Discussion

### Phenotypic differentiation

These experiments are consistent with previously reported rapid evolution between the native and invaded ranges of *C. diffusa* over a timescale of a century (Turner et al. 2014). Under benign common conditions in a field experiment in the naturalized range of the species, *C. diffusa* consistently demonstrated morphological and life-history trait differences with greater growth and vegetative biomass for invasive compared to native individuals. This differentiation occurred even in the more realistic field setting, which allowed for biotic and soil interactions, and some degree of conspecific competition (although these were not experimentally controlled).

As reported in many other studies (Müller-Schärer et al. 2004; Williams 2009; Kumschick et al. 2013), invasive individuals grew faster and were thus larger than natives on average in this common garden study. In a previous study including these and many additional populations, invasive individuals were both larger and produced more seed in a common environment than natives (Turner et al. 2014), a positive relationship which has been demonstrated between size and fitness for other monocarpic species (reviewed in Metcalf et al. 2003). Given that, this result demonstrates a potential for increased fitness, and therefore invasiveness, among North American populations. Additionally, invasive populations demonstrate less variation in size traits across the invaded range (indicated by narrower standard error bars, Fig.3B and F, Figure S4B and C) than native populations, possibly the result of directional selection in the invaded range for larger individuals. However, the populations included here are only a subset of all populations; it is therefore possible that the native source populations that generated the invasive populations are not included, and that trait differences between the groups evolved prior to introduction. If the native source populations are not included in this experiment, which cannot be verified without genotypic information, it is possible that phenotypic difference observed here represents evolved difference among native populations, rather than adaptation to the invaded range per se. However, a glasshouse study that included many more populations reported similar phenotypic differentiation as was observed here (Turner et al. 2014). Ultimately, genotypic information is needed to establish the identity of the source populations for this invasion.

Invasive individuals exhibited delayed maturity, a result that may suggest either an adaptation to a longer growing season or a shift toward bienniality, which was also found in two previous glasshouse common gardens (Turner et al. 2014). Such a shift in reproductive strategies has already been documented in the facultative monocarp houndstongue *Cynoglossum officinale* L., where iteroparity was much more common in the introduced range than in the native range (Williams 2009). We did not detect any difference in mortality between ranges during the first growing season. If this result holds after a second growing season, delayed maturity might tentatively represent a fitness increase in the invaded range. If delayed bolting means the plant is larger at maturity, then it has the potential to produce more flower heads, more seed, and more progeny. To test this hypothesis, additional experiments are needed to link demographic parameters measured at the individual level such as survival and growth to population dynamics.

Although we cannot rule out the existence of maternal effects in the present experiment, it is very unlikely that such effects are the only source of phenotypic differentiation between native and invasive *C. diffusa*. First, previous work reported similar results for collections from natural populations versus those derived from *C. diffusa* plants that were produced from controlled crosses and grown under common conditions in the glasshouse, thereby controlling for maternal effects (Turner et al. 2014). Second, maternal effects in plants are mostly predominant for early traits in the life cycle such as during the germination stage (see Weiner et al. 1997 in *Centaurea stoebe* subsp. *micranthos*) and are thus unlikely to affect traits such as those measured at harvest for which we observed significant differentiation between native and invasive populations. Additionally, results from the control treatment in this experiment are qualitatively similar to those of previous glasshouse experiments (Turner et al. 2014).

All together, these results suggest that the observed phenotypic differentiation between the native and invaded ranges has a genetic basis. To understand whether or not this differentiation is due to differences in local adaptation to environmental gradients between the ranges (Hypothesis 1a), we assessed the relationship between environment and plant traits. In the present study, individuals from the native and invaded ranges of *C. diffusa* varied not just in phenotype, but in how that phenotype relates to abiotic environmental variation. The composite environmental covariate used here from the experimental PCA had greater explanatory power than latitude alone; latitude contributed only 5% to the variance of PC1 (*not shown*). Native populations demonstrated a significant relationship with this abiotic environmental gradient for several size and life-history traits, which was typically nonsignificant in invasive populations. This may indicate that while native populations are locally adapted to their environments, invasive populations have yet to fully adapt to the invaded range, although the climatic niches inhabited by these populations largely overlap along the two main axes of climate variation in the PCA of sampled populations (Fig.2, Table S2).

### Water limitation response and resource allocation trade-offs

Many attempts to explain invasion success have invoked trade-offs between growth or resource allocation and tolerance to stress characteristic of the native range, such as biotic (Blossey and Notzold 1995; Joshi and Vrieling 2005) or abiotic stresses (Bossdorf et al. 2005; He et al. 2010). For instance, a pattern consistent with a trade-off between growth and tolerance to drought stress has been shown in *Ambrosia artemisiifolia* (Hodgins and Rieseberg 2011). In terms of water limitation response (Hypothesis 1b), we found no evidence of variation in resource allocation and water limitation tolerance between the ranges in the present study, in contrast to Turner et al. (2014). That said, only two of 13 measured traits demonstrated a significant effect of treatment, suggesting a limited impact of water addition on plants in this case. Such limited effect may arise from the difficulty of controlling water supply in an open field setting; because we did not measure soil moisture directly, it is impossible to rule this out as a cause for the weak treatment effect. For instance, it may be explained by sufficient natural rainfall during the duration of the experiment. Alternatively, trade-offs with drought stress reported previously might be an artifact of having conducted the drought evaluations in the glasshouse (e.g., plants might have become root bound) and therefore unlikely to materialize under field conditions.

### Environmental gra dients, plasticity, and niche expansion

In the absence of evidence for local adaptation to the environment in the invaded range, invasive populations may have adopted a Jack-of-all-trades, environmental generalist, strategy (Hypothesis 2). Our results showing a lack of correlation between environmental gradient in the invasive range and phenotype, in other words, that phenotype in the invaded range is largely insensitive to environment, is the result one would expect under a Jack-of-all-trades scenario (Richards et al. 2006). Fitness homeostasis due to Jack-of-all-trades type plasticity has been reported from the *Centaurea* genus before, across other types of environmental resource gradients; both when comparing several highly successful invasive to noninvasive congeners across water and phosphorous gradients (Muth and Pigliucci 2007), and between invasive and noninvasive cytotypes of *C. stoebe* s.l. across study site climatic and soil conditions (Hahn et al. 2012). In *C. diffusa*, the robust (and potentially more fit) performance of invasive populations is retained across environmental conditions, but also across different stress treatments (here, water limitation, but in Turner et al. (2014) across several other stresses as well). Alternatively, this may indicate that invasive populations have adapted along an environmental gradient not seen in the native range. In fact, invasive phenotypes have a significant relationship to the environmental gradient used here for only two traits, bolting probability and wilting date, and for both traits, although the slope is weaker, the opposite trend was seen between ranges. It should be noted that the composite environment observed here does not necessarily vary monotonically.

Although niche-based distribution models assume that invasive species’ responses to environmental gradients (i.e., their ecological niche) are conserved between ranges (Peterson 2003), some studies suggest that responses can vary among the ranges (Broennimann et al. 2007; Fitzpatrick et al. 2007) although this is rare for terrestrial plant invaders (Petitpierre et al. 2012). The flat relationship between phenotype and local environmental conditions seen in the invaded range of *C. diffusa* fails to support a local adaptation hypothesis (1a) and is a pattern which may be common in the genus (Hahn et al. 2012; Broennimann et al. 2014). This contrasts sharply with the strong, adaptive, latitudinal, or altitudinal clines in traits related to growth, phenology, and life history, which appear to be common in introduced plants (Huey et al. 2000; Maron et al. 2004; Alexander et al. 2009; Colautti et al. 2009). Nor is it the case that *C. diffusa* was too recently introduced to develop a clinal relationship; this pattern is apparent in *Lythrum salicaria*, introduced at approximately the same time (Colautti et al. 2010). Novel abiotic conditions in the invaded range (such as those experienced in the areas of putative niche expansion seen in occurrence PCA for *C. diffusa*), biotic interactions (Keane & Crawley 2002), or genetic composition (Ellstrand and Schierenbeck 2000; Bossdorf et al. 2005; Taylor & Keller 2007) could alter or limit plant responses to similar environmental gradients between ranges (Alexander et al. 2009). There is little evidence that reductions in genetic variation have limited local adaptation in the invaded range as comparable genetic diversity exists in both ranges (Hufbauer and Sforza 2008). The lack of apparent local adaptation is consistent with the prominence of “general-purpose” genotypes in the invaded range, which have a plastic, robust performance across environments and have not been selected to specialize in any particular environment (Hypothesis 2; Baker and Stebbins 1965; Hahn et al. 2012).

While this analysis represents a coarse assessment and necessitates many caveats, our observation of a difference in climatic space occupied by *C. diffusa* in the native and invaded ranges is meant as a suggestion of how such patterns might be reflected in the larger global context, and as a starting point for further investigation. Detecting such shifts where they occur is important, both for predictive management and because invasions characterized by niche expansion deserve increased scrutiny, to help us understand when this is likely to occur in other situations, such as climate change (Guggisberg et al. 2012; Petitpierre et al. 2012). Realized niche shifts cannot alone indicate adaptation into novel habitats (i.e., change in the fundamental niche) in the invaded range. Ordination analysis, though likely to quantify niche overlap more accurately overall than ecological niche modeling alone (Broennimann et al. 2014), brings with it several caveats (Guisan et al. 2007). First, occurrence data of the type used in the occurrence PCA (which does not include absence data) very likely underestimate the distribution of the species or alternately oversample some areas in either range. It is possible that collection or reporting effort may vary between ranges and therefore bias the results seen here. The pattern observed here may in part be due to the highly clumped nature of the GBIF occurrence data, which may oversample some locations (though note that proximity in climate space does not necessarily imply proximity in geographic space). Second, this analysis makes no attempt to assess the availability of analog versus nonanalog habitats between the two ranges and can therefore only suggest the possibility of the evolution of a climatic niche expansion. Finally, we can only assess the realized, not the fundamental niche of this species using occurrence data. Biotic interactions and dispersal may limit the realized niche in the native range, and these limiting factors may shape occurrences in the two ranges differently. However, coupled with evidence of genetically based phenotypic change, the putative shift in the realized niche of *C. diffusa* in the invaded range suggests that phenotypic change may have coincided with the evolution of increased physiological tolerance (Guisan et al. 2014). Though rare (Petitpierre et al. 2012), niche shifts or expansions have been demonstrated in some invasive plants. For example, some populations of *Pinus taeda,* grown in replicated common gardens outside its native range, were more invasive in climate niche spaces distinct from those of their native source range (Zenni et al. 2014a). Perhaps the best supported example of a realized niche expansion occurring in the invaded range of a plant is from the closely related spotted knapweed. Spotted knapweed (*Centaurea stoebe* subsp. *micranthos*) has expanded its realized niche, demonstrated from two replicated spatio-temporal invasion routes through North America, to eventually encompass wetter, drier, and warmer conditions than those experienced in the native range (Broennimann et al. 2014).

### Hybridization and phenotypic plasticity

Genetic changes induced by inter- or intraspecific hybridization have been hypothesized to promote invasiveness (Schierenbeck and Ellstrand 2009). *Centaurea diffusa*, a diploid, has a history of hybridization with diploid spotted knapweed, *C. stoebe* subsp. *stoebe* L., which is also native to eastern Europe (Blair and Hufbauer 2010; Blair et al. 2012; Lai et al. 2012; Mráz et al. 2012a). Although *C. stoebe* subsp. *stoebe* does not occur in North America (Treier et al. 2009; Blair and Hufbauer 2010), the tetraploid form (*C. stoebe* subsp. *micranthos* [Gugler] Hayek, sometimes referred to as *C. maculosa*) has invaded the United States with dramatic success. Hybrids between *C. diffusa* and *C. stoebe* subsp. *stoebe* have been reported in both ranges (Blair et al. 2012; Lai et al. 2012). The lack of reestablishment of adaptation to an environmental gradient among invasive populations could be the result of common hybrid ancestry from *C. stoebe* subsp. s*toebe* throughout the invaded range. Indeed, hybridization may play a role in the prominence of plastic, stress-tolerant “general-purpose” genotypes (Schierenbeck and Ellstrand 2009; Blair et al. 2012; Parepa et al. 2014). Although the process of invasion alone, by exposing populations to extreme environmental changes, can result in the rapid evolutionary increase of plasticity in the early stages of invasion (Bock et al. 2015; Lande 2015), this benefit may be transient, and selection may then favor a locally adapted fixed phenotype if there is a cost associated with maintaining plasticity (Lande 2009, 2015). Heterosis resulting from hybridization, however, is known to stabilize fitness across environments (Lippman & Zamir 2007; Bock et al. 2015), and this stabilization could be observed as the loss of environmental adaptation in the invaded range and may also enhance invasiveness by providing an advantage over parental taxa (Burke and Arnold 2001). We do not know the level of introgression of the majority of populations used in this study (but see Table S1). Further comparisons, including comprehensive genomic studies of admixture, are thus needed to assess the extent of introgression and its impact on the performance of invasive populations of *C. diffusa*.

## Conclusion

The invaded range of *C. diffusa* is dominated by genetically and phenotypically differentiated plants, which are larger, with delayed maturity, and a more generalist relationship to climate, relative to the native range. While local adaptation along a resource allocation trade-off for water limitation tolerance is equivocal, local adaptation to abiotic climatic conditions is evident in the native range. However, invasive populations do not show such relationship between phenotypic variation and climate. Instead, a plastic, generalist strategy may have been favored in the invaded range, resulting in the expansion of the species into a greater diversity of environments. This could make climatic niche-based predictive distribution models built on data from the native range potentially uninformative for this species (Broennimann et al. 2007). Future work will attempt to address the role of hybridization in the production of hugely successful plastic phenotypes in the invaded range of *C. diffusa*.

## Data Accessibility

Data available from the Dryad Digital Repository: http://dx.doi.org/10.5061/dryad.doi:10.5061/dryad.60p5d. Code for analysis and figures are available on GitHub: https://github.com/kgturner/FranceCG/.

## Supporting Information

**Data S1.** Explicit trade-off models of tolerance to water limitation in *Centaurea diffusa*. **Figure S1.** Additional figures from the principal components analysis of environmental variables for *C. diffusa* populations used in the field experiment. **Figure S2.** Correlation among plant traits measured in field common garden, using Kendall’s *tau* statistic. **Figure S3.** Additional examples of morphological and stress tolerance trait divergence among *Centaurea diffusa* in the field experiment for traits with significant origin or origin-by-environment terms in range differentiation models. **Figure S4.** Principal components analysis of environmental variables for all *C. diffusa* geo-referenced occurrences recorded in GBIF.org. **Figure S5.** Principal components analysis of environmental variables for a subset of *C. diffusa* geo-referenced occurrences recorded in GBIF.org. **Table S1**. *Centaurea diffusa* sampled population information. **Table S2.** Abiotic environmental data variables used in principal components analyses (Hijmans et al. 2005). **Table S3.** Test statistics for all traits measured in the field common garden, from range differentiation models of phenotype of *Centaurea diffusa*. **Table S4.** Parameter estimates of fixed effects from range differentiation models of *Centaurea diffusa* grown in field common garden experiment which included a significant origin term or significant interaction between origin and environment.
