# Supplementary material for: Adaptive plasticity and niche expansion in an invasive thistle
Source: Ecol Evol. 2015 Jul 14;5(15):3183–97. doi: 10.1002/ece3.1599 (PMC4559060; doi:10.1002/ece3.1599)
Supplement: Supplementary file 1 [file ece30005-3183-sd1.docx]

*Supplementary materials*

**Data S1** Explicit trade-off models of tolerance to water limitation in *Centaurea diffusa*.

Additional trade-off models explicitly tested for a trade-off between performance in benign water addition conditions and tolerance to water limitation (as with the extreme drought treatment in Turner et al. 2014). In these models, a population mean measure of performance in the benign control treatment (log transformed population mean shoot mass) and its interaction with origin were included in models using only individuals in the water limitation treatment, along with all of the terms included in the range differentiation models (Trait ~ Origin * Control population mean mass + Environment + (1 | Population/Maternal line)). A significant interaction would indicate a different relationship between performance under benign and stressful conditions between the two origins.

**Figure S1** Additional figures from the principal components analysis of environmental variables for *C. diffusa* populations used in the field experiment. Axis 1 and 2 are discussed within the main text. (a) Scree plot of eigenvalues for the top ten principal components. (b) PC1 versus PC3, with bioclimatic variables labeled (explained in Table S2). Shaded area represents 95% confidence ellipse, using k-means clustering and assuming two blind clusters (only a single population falls in cluster 2). (c) PC1 versus PC3. Axis 3 explains 14% of variance, and is correlated with mean temperature of warmest quarter, annual mean temperature, and maximum temperature of the warmest month (BIO10, BIO1, BIO5; Table S2).


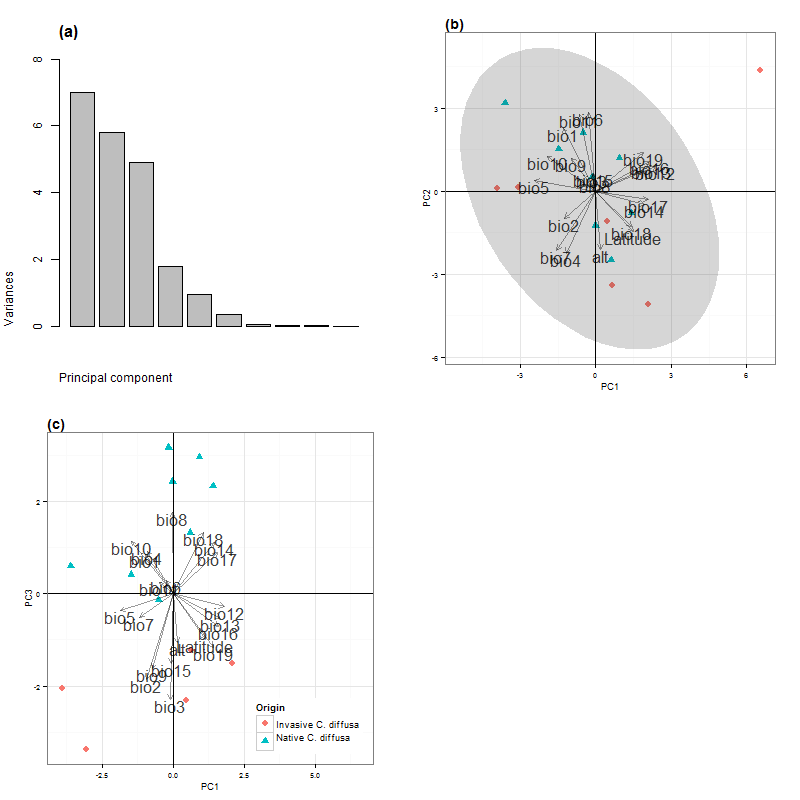


**Figure S2:** Correlation among plant traits measured in field common garden, using Kendall’s *tau* statistic. Some traits which were measured on only a subset plants (such as bolting date) are not included. Repeated measures are presented as separate traits, followed by the time point (for example, “Leaf.count.t1” is leaf number measured at time point 1).**
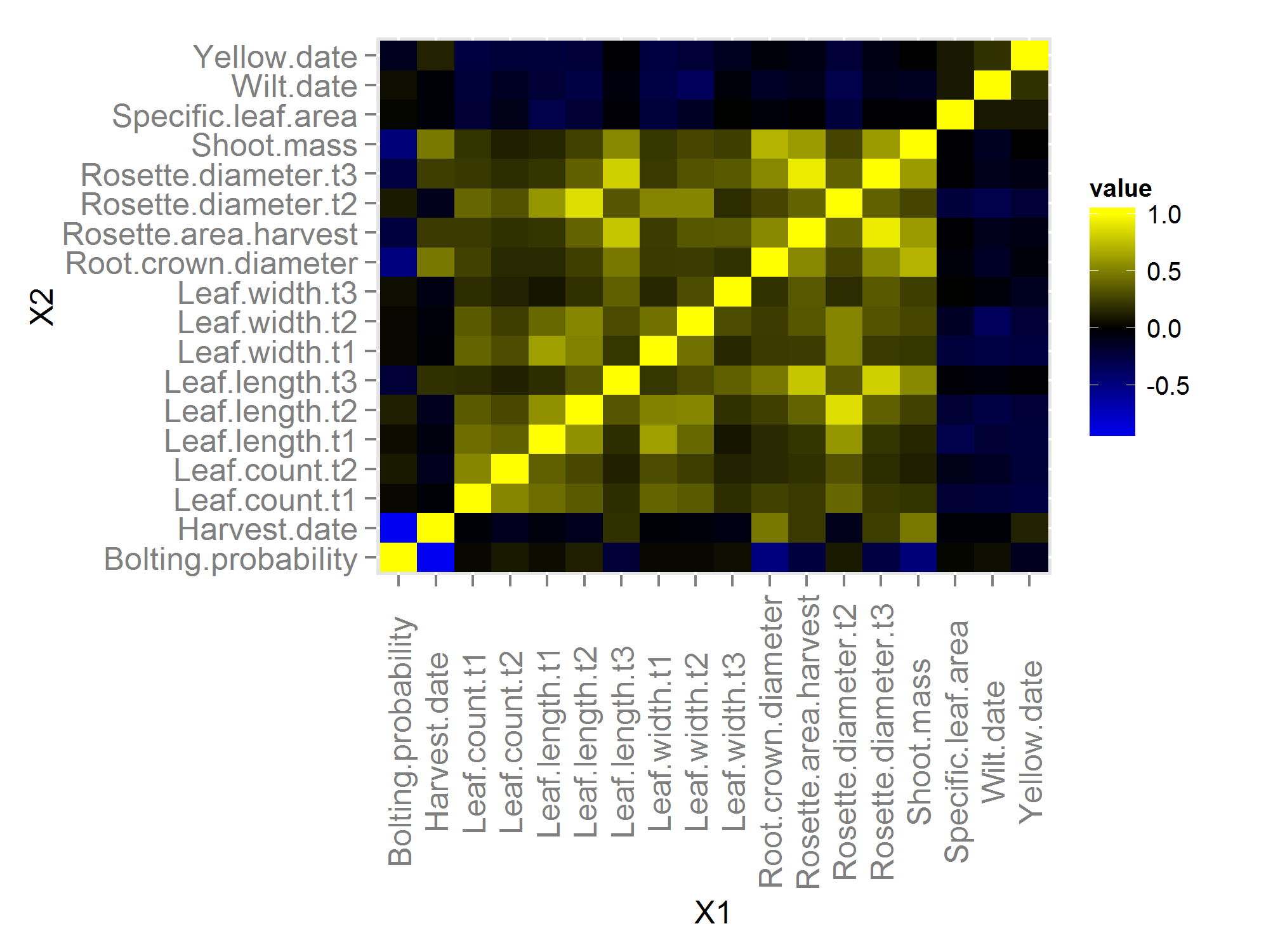
**

**Figure S3:** Additional examples of morphological and stress tolerance trait divergence among *Centaurea* *diffusa* in the field experiment for traits with significant origin or origin-by-environment terms in range differentiation models. Environmental gradient is represented in each figure by PC1 of sampled population locations. All figures are from observed data; model parameters are described in Table 1 and Table S4. Shaded area represents standard error. (a) Population mean basal leaf number for three time points (>3 cm in length; significant origin term). (b) Population mean width of longest leaf along the environmental gradient, for three time points (significant origin-by-environment term). (c) Population mean rosette area at harvest along the environmental gradient (significant effect of origin-by-environment and treatment terms). (d) Population mean wilt date, among plants which wilted, along the environmental gradient (significant effect of origin-by-environment and treatment terms). (e) Population mean yellowing date, among plants which yellowed, along the environmental gradient (significant origin-by-environment term).


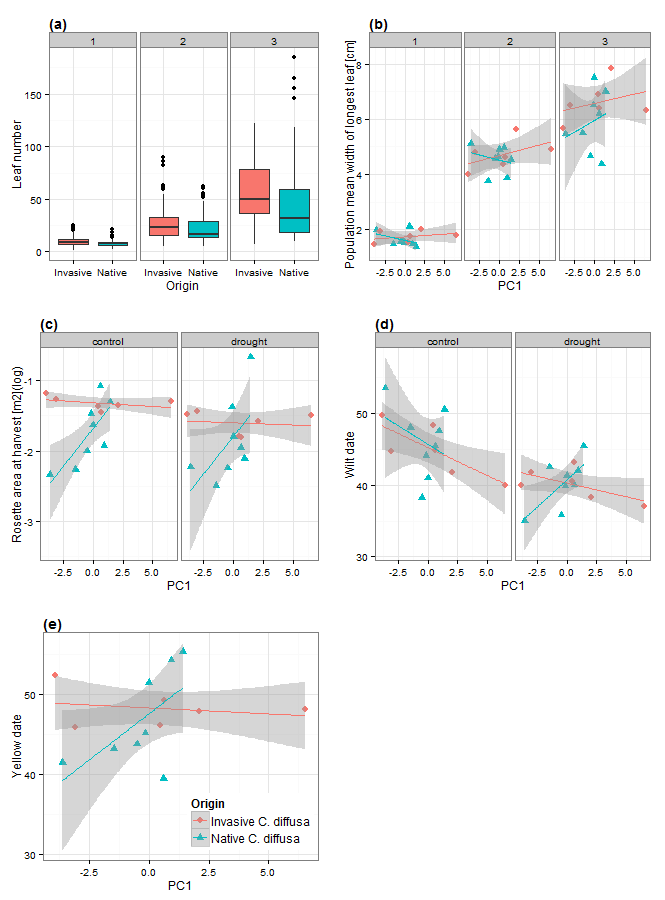


**Figure S4:** Principal components analysis of environmental variables for all *C. diffusa* geo-referenced occurrences recorded in GBIF.org. Axis 1 and 2 are discussed within the main text. (a) Scree plot of eigenvalues for the top ten principal components. (b) PC1 versus PC2, with bioclimatic variables labeled (explained in Table S2). (c) PC1 versus PC3, including 99% confidence ellipse. Axis 3 explains 15% of variance, and is correlated with mean temperature of warmest quarter, annual mean temperature, and mean temperature of wettest quarter (BIO10, BIO1, BIO8; Table S2). **
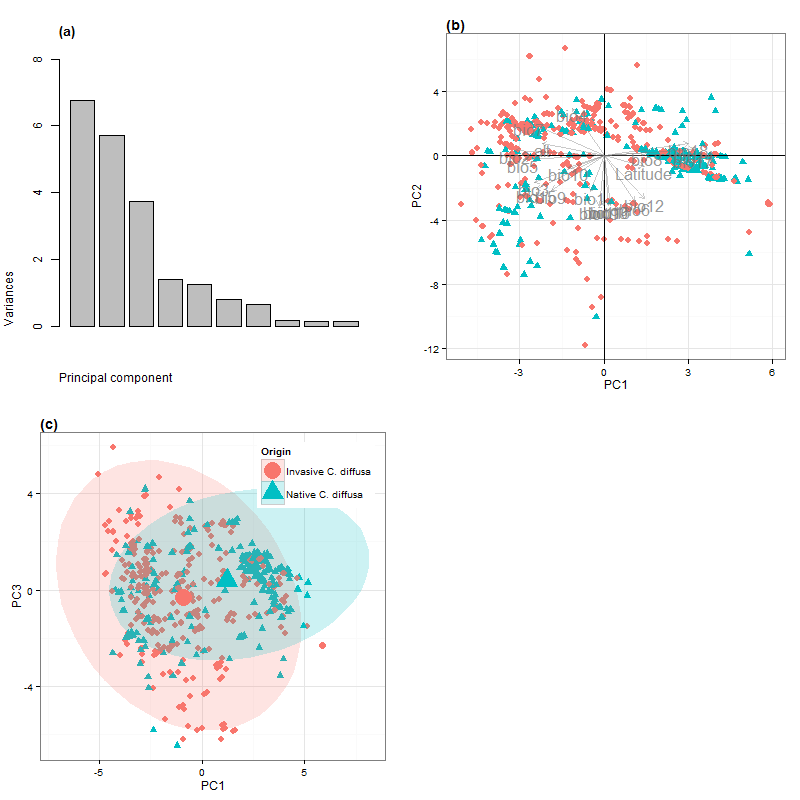
Figure S5:** Principal components analysis of environmental variables for a subset of *C. diffusa* geo-referenced occurrences recorded in GBIF.org. Because the GBIF data used here may not be error free, data was subdivided to include only populations within two standard deviations of the mean of PC1 and PC2 (613 populations) in the larger analysis of occurrence data. Axis 1 explains 35% of variance and correlated with the same top three bioclimatic variables as in the larger occurrence data set (BIO17, BIO14, BIO2; Table S2). Axes 2 and 3 explain 22% and 19% of variance respectively. Relative to the larger occurrence data set, top loadings for Axis 2 and Axis 3 are reversed (Axis 2: BIO11, BIO1, BIO6; Axis 3: BIO19, BIO16, BIO13). (a) Scree plot of eigenvalues for the top ten principal components. (b) PC1 versus PC2. Centroid of niche marked by large point, and significantly varied between ranges (between group inertia: 9.31%; P=0.001 ).(c) PC1 versus PC3. (b) and (c) include 99% confidence ellipses.

**
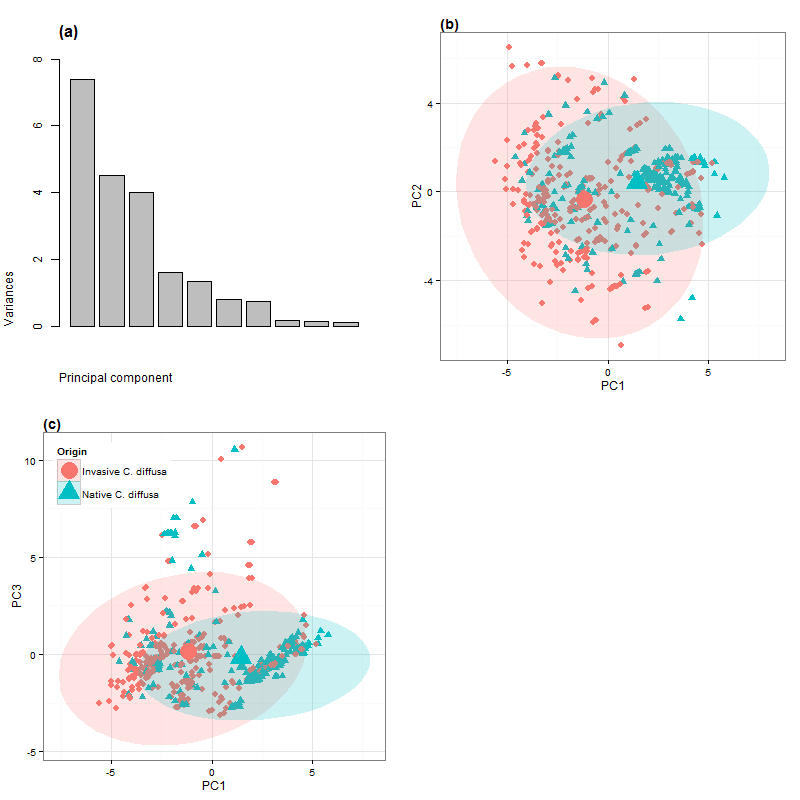
**

**Table S1**: *Centaurea diffusa* sampled population information.

| **Population ID** | **Origin** | **Collection year** | **Latitude** | **Longitude** | **PC1** | **Voucher Accession** |
| --- | --- | --- | --- | --- | --- | --- |
| BG001 | Native C. diffusa | 2008 | 43.38194 | 28.4575 | 0.9307 | V236763 |
| GR001 | Native C. diffusa | 2008 | 40.15667 | 22.54806 | -3.6021 | V236766 |
| GR002 | Native C. diffusa | 2008 | 40.62139 | 23.07861 | -1.4673 | V232702, V236769 |
| HU001 | Native C. diffusa | 2008 | 47.64194 | 18.7825 | 1.4131 | V236764 |
| RO001 | Native C. diffusa | 2008 | 44.11028 | 28.63694 | -0.1482 | V236767 |
| RO005 | Native C. diffusa | 2005 | 45.49786 | 27.91181 | -0.0064 | V232686, V232683 |
| RU008* | Native C. diffusa | 2006 | 44.05 | 43.06 | 0.6099 | V232687 |
| TR001* | Native C. diffusa | 2008 | 41.75111 | 27.24778 | -0.4996 | V236765 |
| CA001 | Invasive C. diffusa | 2008 | 49.01494 | -122.882 | 6.5318 | V232677- V232679, V232704 - V232706 |
| CA009 | Invasive C. diffusa | 2010 | 49.2961 | -118.474 | 0.6531 |  |
| CA010 | Invasive C. diffusa | 2010 | 49.32018 | -119.630 | 2.0793 |  |
| US001 | Invasive C. diffusa | 2008 | 45.61523 | -120.788 | -3.0682 | V232694 - V232697 |
| US002 | Invasive C. diffusa | 2008 | 46.18227 | -118.826 | -3.8945 | V232669 - V232671 |
| US003 | Invasive C. diffusa | 2008 | 46.60415 | -116.642 | 0.4684 |  |

Vouchers are located in the UBC Herbarium. PC1 values for each population are taken from the experimental principal components analysis, described in the main text. * Indicates additional information on levels of introgression in that population, as follows. Population RU008 was verified as diploid (RU119; Blair et al., 2012) and 0 of 4 individuals from that population were determined to be introgressed from AFLP analysis (Blair and Hufbauer, 2010). An individual from population TR001 was used by Lai et al. (2012), to determine approximate level of introgression when compared to a tetraploid individual of *Centaurea stoebe* ssp. *micranthos*. In that study, the individual from TR001 was found to have a lower extent of introgression than an individual from the invaded range.

**Table S2:** Abiotic environmental data variables used in principal components analyses (Hijmans et al., 2005).

| **Bioclim variable** | **Code** |
| --- | --- |
| Annual mean temperature | BIO1 |
| Mean diurnal temperature range (mean of monthly (max temp – min temp)) | BIO2 |
| Isothermality (BIO2/BIO7)*100 | BIO3 |
| Temperature seasonality (standard deviation*100) | BIO4 |
| Max temperature of warmest month | BIO5 |
| Min temperature of coldest month | BIO6 |
| Temperature annual range (BIO5-BIO6) | BIO7 |
| Mean temperature of wettest quarter | BIO8 |
| Mean temperature of driest quarter | BIO9 |
| Mean temperature of warmest quarter | BIO10 |
| Mean temperature of coldest quarter | BIO11 |
| Annual precipitation | BIO12 |
| Precipitation of wettest month | BIO13 |
| Precipitation of driest month | BIO14 |
| Precipitation seasonality (coefficient of variation) | BIO15 |
| Precipitation of wettest quarter | BIO16 |
| Precipitation of driest quarter | BIO17 |
| Precipitation of warmest quarter | BIO18 |
| Precipitation of coldest quarter | BIO19 |
| Altitude | ALT |
| Latitude | LAT |

Data taken from current conditions (interpolations of observed data, representative of years 1950-2000) from ~1km tiles numbers 6, 7, 11,12,13,15,16,17,18.

**Table S3:** Test statistics for all traits measured in the field common garden, from range differentiation models of phenotype of *Centaurea diffusa*.

|  | *Fixed effects* | | | | *Random effects* | | |
| --- | --- | --- | --- | --- | --- | --- | --- |
|  | Origin | Env | Origin*  Env | Treatment | Population | Maternal lines | Repeat measure |
| *Trait* | **χ2 (df) *P*** | **χ2 (df) *P*** | **χ2 (df) *P*** | **χ2 (df) *P*** | **χ2 (df) *P*** | **χ2 (df) *P*** | **χ2 (df) *P*** |
| Number of basal leavesǂ | 5.82 (1) * | 0.49 (1) | 0.03 (1) | 0.12 (1) | 0.81 (1) | 0 (1) | 552.87 (3) *** |
| Length of longest leaf | 3.14 (1) . | 0 (1) | 0.39 (1) . | 2.13 (1) | 7.03 (1) ** | 12.76 (1) *** | 134.42 (3) *** |
| Width of longest leaf | nt | nt | 8.50 (1) ** | 0.82 (1) | 1.02 (1) | 0.85 (1) | 132.38 (3) *** |
| Rosette diameter | 3.70 (1) . | 0 (1) | 0.39 (1) . | 1.45 (1) | 4.58 (1) *£ | 13.03 (1) *** | 161.68 (3) *** |
| Root crown diameter | nt | nt | 9.88 (1) ** | 0.82 (1) | 14.89 (1) *** | 16.33 (1) *** | --- |
| Rosette area at harvest | nt | nt | 8.35 (1) ** | 5.23 (1) * | 24.23 (1) *** | 3.16 (1) . | --- |
| Bolting probability | nt | nt | 37.19 (1) *** | 0.06 (1) | 0 (1) | 0 (1) | --- |
| Mortality | 0.12 (1) | 1.14 (1) | 0.01 (1) | 0.66 (1) | ǂ1.30 (1) | 0 (1) | --- |
| Specific leaf area | 2.49 (1) | 0.05 (1) | 1.05 (1) | 0.02 (1) | 1.30 (1) | 0 (1) | --- |
| Bolt date | nt | nt | 9.34 (1) ** | 0.07 (1) | 4.84 (1) *£ | 0 (1) | --- |
| Shoot mass | nt | nt | 14.44 (1) *** | 1.71 (1) | 14.82 (1) *** | 9.71 (1) ** | --- |
| Wilt date | nt | nt | 6.28 (1) * | 21.42 (1) *** | 4.76 (1) *£ | 0 (1) | --- |
| Yellow date | nt | nt | 25.89 (1) *** | 0.46 (1) | 4.45 (1) *£ | 0 (1) | --- |

Results are presented from restricted maximum likelihood (REML) models. Significance of term indicated by symbol: ., *P* < 0.1; *, *P* < 0.05; **, *P* < 0.01; ***, *P* < 0.001. Env = environment term. (df) = degrees of freedom. χ^2^ = chi-squared test statistic. *Nt* = not tested because of significant interaction term. ǂData scaled when necessary to improve model performance. £ Non-significant after FDR correction.

**Table S4:** Parameter estimates of fixed effects from range differentiation models of *Centaurea diffusa* grown in field common garden experiment which included a significant origin term or significant interaction between origin and environment.

| *Trait* | **Distribution, model** | **Fixed effect** | **Estimate** | **SE** | **Test statistic** | ***P*** |
| --- | --- | --- | --- | --- | --- | --- |
| Number of basal leaves | Gaussian (scaled), REML | Invasive mean (intercept) | 0.37 | 0.02 | 15.86 | *** |
|  |  | Difference between origins | -0.07 | 0.03 | -2.42 | * |
|  |  |  |  |  |  |  |
| Leaf width | Gaussian, REML | Invasive mean (intercept) | 3.32 | 0.11 | 30.15 | *** |
|  |  | Difference between origins | -0.31 | 0.14 | -2.16 | * |
|  |  | Invasive slope along environmental cline | 0.07 | 0.04 | 1.86 | . |
|  |  | Difference in slopes along environmental cline | -0.22 | 0.07 | -3.00 | ** |
|  |  |  |  |  |  |  |
| Root crown diameter | Gaussian (log_e_), REML | Invasive mean (intercept) | 3.18 | 0.11 | 28.91 | *** |
|  |  | Difference between origins | -0.23 | 0.15 | -1.60 |  |
|  |  | Invasive slope along environmental cline | -0.02 | 0.03 | -0.65 |  |
|  |  | Difference in slopes along environmental cline | 0.23 | 0.07 | 3.27 | ** |
|  |  |  |  |  |  |  |
| Rosette area at harvest | Gaussian (log_e_), REML | Invasive mean (intercept) | -1.36 | 0.12 | -11.53 | *** |
|  |  | Difference between origins | -0.31 | 0.15 | -2.03 | . |
|  |  | Invasive slope along environmental cline | -0.01 | 0.04 | -0.39 |  |
|  |  | Difference between treatments | -0.15 | 0.07 | -2.29 | * |
|  |  | Difference in slopes along environmental cline | 0.22 | 0.08 | 2.92 | * |
|  |  |  |  |  |  |  |
| Shoot mass | Gaussian (log_e_), REML | Invasive mean (intercept) | 3.99 | 0.19 | 20.50 | *** |
|  |  | Difference between origins | -0.53 | 0.26 | -2.05 | . |
|  |  | Invasive slope along environmental cline | -0.05 | 0.06 | -0.89 |  |
|  |  | Difference in slopes along environmental cline | 0.54 | 0.13 | 4.30 | ** |
|  |  |  |  |  |  |  |
| Bolting probability | Binomial, REML | Invasive mean (intercept) | -0.94 | 0.24 | -3.93 | *** |
|  |  | Difference between origins | 0.91 | 0.31 | 2.93 | ** |
|  |  | Invasive slope along environmental cline | 0.19 | 0.09 | 2.16 | * |
|  |  | Difference in slopes along environmental cline | -1.11 | 0.22 | -4.99 | *** |
|  |  |  |  |  |  |  |
| Bolt date | Poisson, REML | Invasive mean (intercept) | 3.89 | 0.03 | 133.95 | *** |
|  |  | Difference between origins | -0.02 | 0.04 | -0.46 |  |
|  |  | Invasive slope along environmental cline | 0.003 | 0.01 | 0.39 |  |
|  |  | Difference in slopes along environmental cline | 0.04 | 0.01 | 3.04 | ** |
|  |  |  |  |  |  |  |
| Wilt date | Poisson, REML | Invasive mean (intercept) | 3.80 | 0.02 | 210.06 | *** |
|  |  | Difference between origins | 0.02 | 0.02 | 0.80 |  |
|  |  | Invasive slope along environmental cline | -0.02 | 0.01 | -2.65 | ** |
|  |  | Difference between treatments | -0.11 | 0.02 | -4.60 | *** |
|  |  | Difference in slopes along environmental cline | 0.04 | 0.01 | 2.49 | * |
|  |  |  |  |  |  |  |
| Yellow date | Poisson, REML | Invasive mean (intercept) | 3.88 | 0.02 | 246.85 | *** |
|  |  | Difference between origins | -0.002 | 0.02 | -0.11 |  |
|  |  | Invasive slope along environmental cline | -0.003 | 0.01 | -0.46 |  |
|  |  | Difference in slopes along environmental cline | 0.08 | 0.02 | 5.01 | *** |

Test statistics are presented from range differentiation models, described in Table S3. Results are presented from restricted maximum likelihood (REML) models. For Gaussian distributed models, T values are reported for the test statistic; for Binomial and Poisson distributed models, Z ratios are reported. Models include all significant terms. Significance of term indicated by symbol: ., *P* < 0.1; *, *P* < 0.05; **, *P* < 0.01; ***, *P* < 0.001.
